# Feasibility of Producing Core-Shell Filaments through Fused Filament Fabrication

**DOI:** 10.3390/polym13234253

**Published:** 2021-12-04

**Authors:** Alexandru Sover, Vasile Ermolai, Ashok M. Raichur, Romeo Ciobanu, Mihaela Aradoaei, Nicolae Lucanu

**Affiliations:** 1 Department of Technology, Technical Faculty, Ansbach University of Applied Sciences, 91522 Ansbach, Germany; 2 Department of Materials Engineering, Indian Institute of Science, Bengaluru 560012, India; amr@iisc.ac.in; 3 Department of Electrical Measurements and Electrotechnical Materials, Gheorghe Asachi Technical University, 700050 Iasi, Romania; rciobanu@ee.tuiasi.ro (R.C.); mihaela.aradoaei@academic.tuiasi.ro (M.A.); nicolae.lucanu@academic.tuiasi.ro (N.L.)

**Keywords:** fused filament fabrication, multi-material, core-shell filament, 3D printed filament

## Abstract

Fused filament fabrication is a technology of additive manufacturing that uses molten thermoplastics for building parts. Due to the convenient shape of the raw material, a simple filament, the market offers a great variety of materials from simple to blends of compatible materials. However, finding a material with the desired properties can be difficult. Making it in-house or using a material manufacturer can be costly and time-consuming, especially when the optimum blend ratios are unknown or new design perspectives are tested. This paper presents an accessible method of producing core-shell filaments using material extrusion 3D printing. The printed filaments are characterised by a polycarbonate (PC) core and acryl butadiene styrene (ABS) shell with three material ratios. Their performance was investigated through printed samples. Additionally, the material mixing degree was studied by varying the extrusion temperature, nozzle feeding geometry, and layer thickness. The influence of all four factors was evaluated using a graphical representation of the main effects. The results showed that a core-shell filament can be processed using a 3D printer with a dual extrusion configuration and that the mechanical properties of the samples can be improved by varying the PC–ABS ratio. This research provides an accessible method for developing new hybrid filaments with a predesigned structure using a 3D printer.

## 1. Introduction

Additive manufacturing (AM) represents a versatile set of technologies that can be used conveniently in product development. All parts are manufactured layer by layer directly from the digital 3D data [1,2]. AM comprises a wide range of technologies, materials, and according to ISO 17296-2: 2015, they can be categorised in the following seven groups vat photopolymerisation (VP), material jetting (MJ), binder jetting (BJ), powder bed fusion (PBF), material extrusion (ME), direct energy deposition (DED), and sheet lamination (SL) [3]. Each of these categories contains more than one manufacturing technology. Due to this fact, there is a wide range of available materials such as plastic, metals, and ceramics in liquid, powder, or solid-state [2,4]. The AM process can produce prototypes, tools, and fully functional parts. Besides engineering applications, AM technologies are interdisciplinary and are used in many fields such as medicine, architecture, archaeology [5,6], and many others.

Fused filament fabrication (FFF) or fused deposition modelling (FDM) is a ME technique that uses molten thermoplastic materials to produce parts. As an advantage, FFF disposes of a wide variety of materials from conventional to high-performance polymers with various equipment designs and performances [7].

A drawback of FFF is that the part’s performance is highly influenced by print orientation. For this reason, printed parts are characterised by high anisotropy, and depending on the load direction or the part’s growth direction (z-direction), the behaviour under stress can vary significantly [8]. This downside has led to the need to develop new specimens that better describe the properties of additively manufactured parts. Watschke et al. (2018) developed such samples to characterise the layer bond quality for ME [9].

The appearance of multi-material extrusion systems introduced new opportunities in 3D printing, boosting the quality of the parts (e.g., by using soluble support structure) or the part’s performance under stress (e.g., by producing multi-material components) [10] and has led to new research in the layer bond formation of parts made of similar and dissimilar materials [11]. Besides these, introducing composite materials (e.g., particle reinforced, short carbon, glass, or aramid fibre reinforced) improved the mechanical properties, giving more stability to the components [8,10,12].

The development of co-extrusion equipment allowed parts’ reinforcement with continuous fibres of glass, aramid, or carbon. This way, the mechanical properties of the component produced via FFF registered a significant performance improvement [7,12,13], but implies the acquisition of new hardware.

Besides co-extrusion printing, the use of core-shell filaments was explored, which can be used on conventional FFF 3D printers.

Peng et al. (2018) studied the impact resistance of 3D printed samples made of a bisphenol-A PC core and an ionomer of partially zinc-neutralised polyethene-co- methacrylic acid shell filament produced via conventional extrusion. They concluded that a simple core-shell filament enhances the impact performance by generating new pathways to energy dissipation [14].

The research was continued and in 2019 by studying the tensile properties and impact resistance of filaments with a PC-ABS blend core, with low-density polyethene (LDPE) and high-density polyethene (HDPE) shell produced via screw extrusion. The results show a change in the mechanical properties from brittle, for the PC-ABS material, to a ductile behaviour for the core-shell materials for the tensile test. As for the impact test, samples made from the same filament led to a significant improvement [15].

Ai et al. (2021) extended the work from [15] by studying the dimensional accuracy of core-shell filaments of bisphenol-A PC, copolymer PC (cPC), or PC-ABS core and HDPE shell produced in the same way. Through a higher solidification point, the core acts as a reinforcement, and the shell, due to the lower solidification temperature, offers increased mobility between printed cores, improving the mechanical properties [16].

Hart et al. (2020) studied a different approach of producing core-shell filaments with a PC core and ABS shell via thermal drawing of a FFF 3D printed preform. The study covered the impact performance of core-shell filaments with different core designs, from a star shape to a simple circle. The study shows that thermal drawing is a potential solution for manufacturing a high-performance filament for FFF [17].

This paper aimed to study the feasibility of producing a FFF core-shell filament for research purposes using a dual-extrusion 3D printer. The study covered the design of the filaments and printing process with a PC core and ABS shell with different material ratios. In addition, through a Taguchi L9 experimental matrix, the influence of other printing process parameters was studied. These were nozzle input geometry, extrusion temperature, and layer height. Overall, the most significant factor over sample strength is the ratio of the materials. The tensile test result showed that the sample’s behaviour could transition from ductile to brittle depending on PC–ABS ratios. Multi-material 3D printing can be an accessible solution for developing new filaments with a specific structure for research purposes.

## 2. Materials and Methods

ABS and PC polymers were considered for printing the filament due to their good compatibility and available PC–ABS blend filament on the market as a point of comparison. Since PC material is tougher than ABS, using it as a core can behave similarly to continuous fibres to reinforce the ABS shell [16]. The resulting filaments were printed as samples with flat orientation and evaluated for the tensile test.

### 2.1. Materials

For 3D printing, the core-shell filaments were chosen: a white colour ABS (i.e., Ultrafuse ABS from BFAS) and a black colour PC (i.e., PolyMax PC from PolyMaker). In addition, a third material was used as a benchmark, a white colour PC-ABS (i.e., PolyMaker PC-ABS). According to the manufacturers’ recommendations, all filaments were dried and kept in a dry storage box throughout the study. The same printing conditions were applied for the printed filaments.

### 2.2. Experimental Setup

As a design of experiments method, a Taguchi L9 matrix was used with four factors and three levels of variation (3^4^). For the tensile tests, the 1BA ISO 527 specimen was considered. This setup resulted from the limited filament produced on the printer’s build plate area.

The considered variables were PC–ABS ratios, material extrusion temperature, nozzle input orifices, and layer thickness, and their levels of variation are presented in Table 1. The other process parameters involved in the sample printing process were kept at constant values and are presented in Table 2.

**Figure 1:**
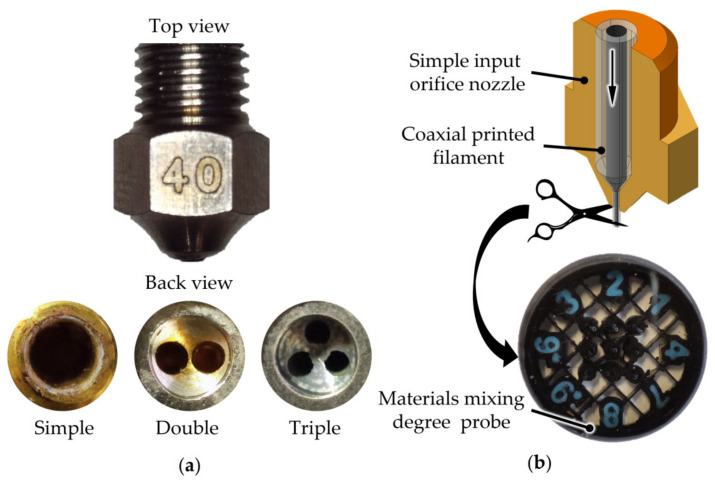
Considered nozzles in the experimental runs. (**a**) Nozzles’ input geometry. (**b**) The method used to take samples of the extruded material.

The variable PC–ABS ratio describes the volume of material of each printed coaxial filament, where the PC is the core and the ABS is the shell. The extrusion temperature range was set between 250 and 260 °C. The lower limit represents the mean melting value of the ABS, and the upper limit the middle melting point of the PC. This range was considered to avoid ABS burning. Nozzle input orifice is a parameter that is related to tool design. For this setup, three types of nozzles were considered: one with a regular geometry with a simple, direct feeding channel, and two with an intermediary wall having two, respectively, three input orifices (Figure 1a). These orifices are drilled holes that start independently and converge at the nozzle’s output channel. This design increases the contact surface area between the material and nozzle, providing even heating (heating the filament from outside-in and inside-out). Due to the increased heating area, material flow and homogeneity were improved [18]. In order to appreciate the influence of nozzle input geometry over the homogeneity, for each run included in the experiment matrix, a sample of extruded material was taken and used for visual inspection (Figure 1b). The last considered parameter is layer thickness. As the nozzles exercise a certain pressure over the material during the deposition, the layer height could influence the material flow [19].

### 2.3. Design and Parametrisation

**Figure 2:**
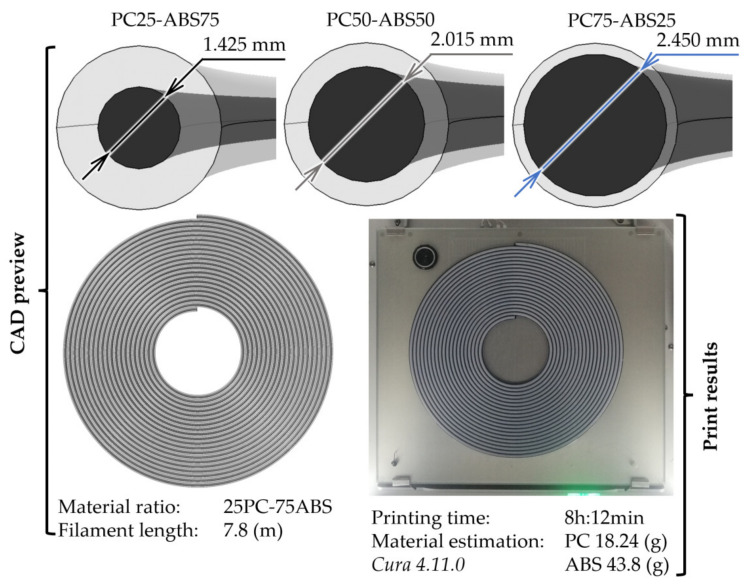
From CAD design to 3D printed core-shell filaments.

The available print area in dual extrusion mode defines the maximum filament length and must be considered in the CAD design (for Ultimaker 3 printer (Utrecht, the Netherlands), the build area is 193 × 193 mm). After defining the limits, the design process is typical, replicable in any CAD software. First, the directory curve is defined as a spiral characterised by revolutions, start and end radius. Then, the generative profiles must be defined and constrained to respect the materials’ ratios. Two sketches are needed, one for the filament’s core, represented by a circle and one for the shell, defined as a ring, with the smaller circle identical to the core diameter. Then, using a CAD sweep function, each body of the core-shell filament is generated. By knowing the outer shell diameter (i.e., 2.85 mm), the core diameter can be calculated by equalising the areas of the cross-sections. The resulting core diameters for each material ratio are shown in Figure 2. In the case of 75PC-25ABS, the material ratios need to be adjusted due to printing limitations. At PC content of 75%, the wall thickness of the ABS shell was 0.19 mm, below the minimum extrusion width of a 0.25 mm nozzle (i.e., 0.2 mm). Therefore, a core ratio of 73.9% was adopted to obtain the 0.2 mm shell thickness. The 75PC-25ABS designation remained unchanged because the PC material volume was reduced by 1.46% from the nominal ratio.

**Figure 3:**
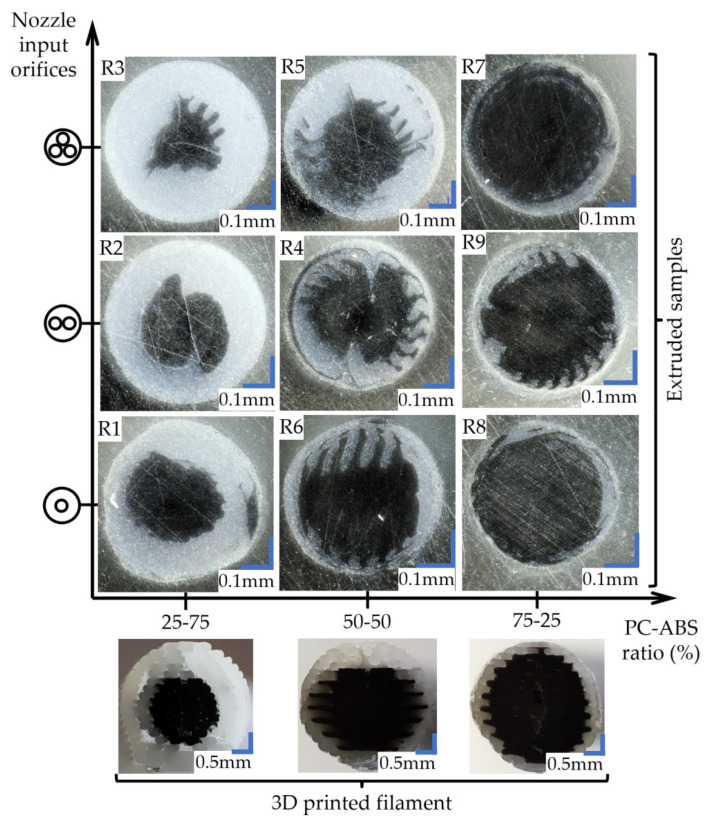
Cross-sectional images of the extruded coaxial filament (300× magnification) and the 3D printed filament cross-section view.

All three filaments were produced with similar parameters settings, with a 0.4 mm nozzle, 0.15 mm layer height, a line width of 0.35 mm, and 35 mm/s printing speed. However, to print the 75PC-25ABS filament’s shell, a smaller nozzle was necessary (i.e., 0.25 mm) and a line width of 0.2 mm. As shown in Figure 3, the resulting core-shell interface does not have a circular profile because the slicing tool alternates (zig-zagging) the core and shell lines to increase horizontal and vertical adhesion between walls, improving the materials’ bond.

### 2.4. Evaluation Methods

An Instron 4411 (Darmstadt, Germany) universal uniaxial testing machine was used with a load cell of 5 kN to evaluate the tensile strength of the samples made of core-shell filament. For each trial covered by the experimental setup, five replicates were tested. The same pattern was used for PC–ABS’s benchmark specimens. Additionally, as a second benchmark, samples made o materials used in printing the core-shell filament were tested. The Minitab 20.4 tool was used as a statistic tool for the data preparation. All reference specimens were produced with the same setup described in Table 2 at 0.15 mm layer height.

To analyse the influence of nozzle feeding geometry and extrusion temperature over the material mixing homogeneity, samples of extruded filament (as presented in Figure 2) were visually evaluated using a Keyence vhx-7000 (Neu-Isenburg, Germany) microscope.

## 3. Results

The results include the study of extruded material homogeneity and the specimens’ tensile strength resulting from the experimental plan with their response discussion. As a result, the main factors of influence were analysed using graphical representation and variance of the responses of the mechanical test. In the end, the fidelity of the linear regression model was validated through empirical trials.

### 3.1. Mixing Degree Performance

The extruded wires were evaluated under a microscope (Figure 3) to appreciate the influence of the extrusion temperature and nozzle input geometry over the PC distribution in the ABS mass.

In the case of samples extruded through the nozzle with simple geometry, it can be observed how the extruded section preserves the structure of the input filament regarding the core-shell concentricity and cross-section aspect. The “zig-zag” pattern was kept between layers even after forcing the material through the nozzle (Figure 3). Furthermore, even with a temperature increase, the proportions of the filament’s cross-section were kept with no noticeable mixing between materials. This aspect suggests that the structure of the 3D printed filament can be maintained during the entire printing process.

Samples extruded through the nozzle with two input orifices showed deformation of the filament’s initial cross-section, giving the PC core more spread in the ABS. In the case of the R2 sample made of 25PC-75ABS and extruded at 255 °C (Figure 3), the two halves of the initial “circular” core glide, resulting in a double snail cam profile. Even so, the core-shell aspect of the materials was preserved for all three extrusion temperatures. Regarding mixing homogeneity, no significant fuse between materials was observed.

The filaments extruded via the nozzle with three input orifices displayed the highest deformation of the PC core from the initial shape. For the 25PC-75ABS material extruded at 260 °C, the core deformed in a triangular shape (Figure 3). The 50PC-50ABS material also presented a high core deformation with an irregular shape, with a higher spread in the ABS mass (Figure 3). Overall, the core-shell form of the materials was maintained for all samples in the group.

### 3.2. Tensile Strength

All samples were tested in the same laboratory conditions with 58% humidity and 23 °C room temperature and at a speed of 5 mm/s. In addition, five replicates were tested for each configuration of the experimental setup, including the benchmark materials.

**Figure 4:**
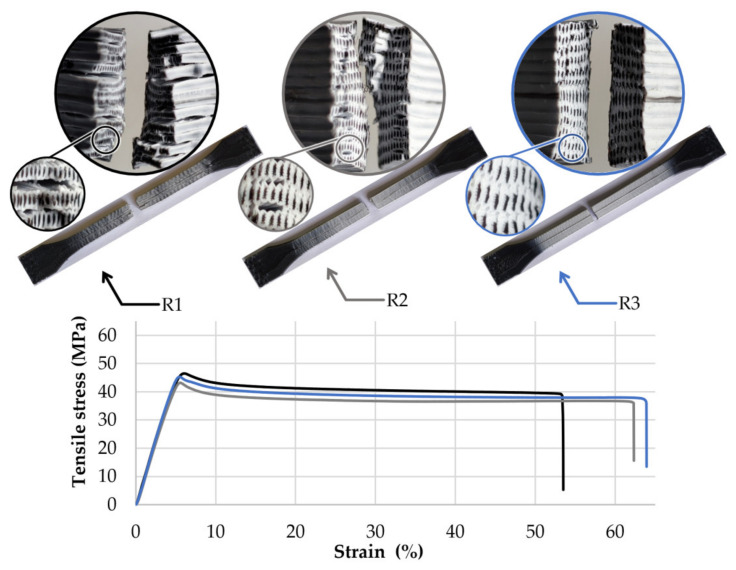
Tensile stress-strain curves, breaking behaviour, and layer structure of the 50PC-50ABS 3D printed filament specimens.

The first group of specimens made of the 25PC-75ABS filament presented similar failure behaviour (Figure 4), characterised by average stress of 44.8 MPa and an average strain of 5.5% before the peak. After reaching the maximum stress, crazing occurred on the gauge length. All samples from this group showed a high elongation with an average of 56.0% before the final break at 35.0 MPa.

**Figure 5:**
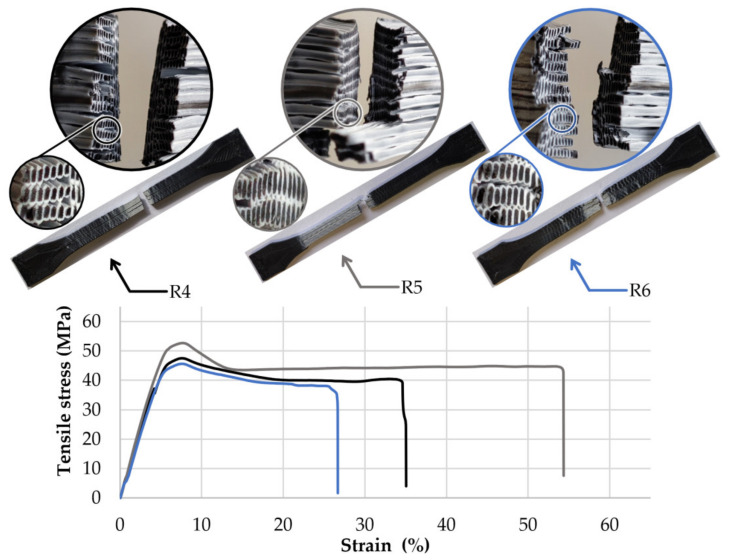
Tensile stress-strain curves, breaking behaviour, and layer structure of the 50PC-50ABS 3D printed filament specimens.

The second group of samples made of 50PC-50ABS filament showed similar breaking behaviour in each set. Their failure modes are presented in Figure 5. Due to the higher content of PC in the filament, the average maximum stress was 49.1 MPa and 7.4% strain before the peak. On average, samples presented a maximum elongation of 23.1% and a yield strength of 38.0 MPa. As soon as the specimens exceeded the maximum stress limit, microfractures of the ABS material whitened the probes’ skin across the gauge length.

**Figure 6:**
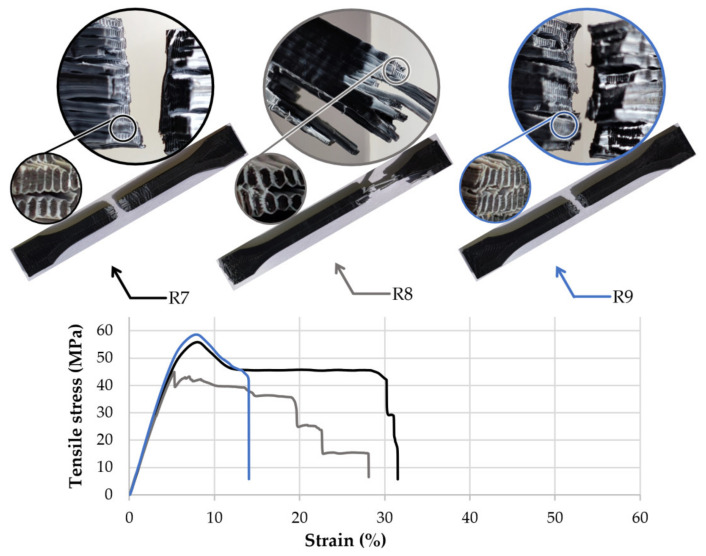
Tensile stress-strain curves, breaking behaviour, and layer structure of the 75PC-25ABS 3D printed filament specimens.

The third group of specimens made of 75PC-25ABS filament (Figure 6) is characterised by high tensile strength of 54.6 MPa with an average elongation of 7.4%. The failure behaviour was akin in each set of samples. In the case of the R8 group with a 0.2 mm layer thickness, sample yield was outside the gauge length and in the same region of the transition radius. Possible explanations are that improper layers fuse due to low extrusion temperature (i.e., 255 °C) correlated with higher layer thickness than other specimens, or residual tension in the area where the samples’ walls merge with the solid layers. The same results were obtained after testing the second set of R8 probes with three replicates. Because of the higher PC content in the filament, all specimens in this group presented a brittle breaking behaviour characterised by higher strength and rapid failure. Again, crazing occurred but in a smaller degree due to the low ratio of ABS.

By analysing the fracture zone of the samples, it can be observed that the core-shell profile of the 3D printed filaments was maintained even after the material was pressed by the nozzle and reshaped in the form of lines (see detail views, Figure 4, Figure 5 and Figure 6).

**Figure 7:**
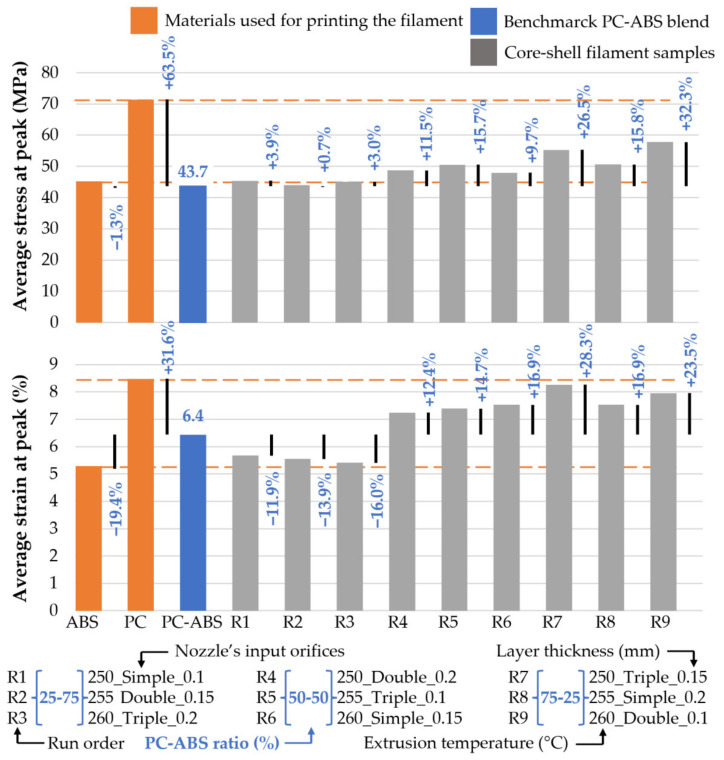
Average tensile stress and strain at the peak of the experimental run specimens referred to the PC-ABS benchmark blend.

After the mechanical tests, the raw data were used to calculate the average stress and strain at peak for each group of samples, including the benchmark probes and the materials used to produce the 3D printed filaments. The results are relevant, presenting minor standard deviations. Overall, stress averages display a standard deviation included in the range (0.59, 2.32) and the strain whiting the interval (0.06, 1,18). The average results were analysed graphically using clustered column charts (Figure 7).

In terms of tensile strength, load capacity was augmented by increased PC content in the 3D printed filament. If in the 25PC-75ABS group, the capacity to take loads increased by 3.9% (R1 set made with a 0.1 mm layer height) compared with the benchmark PC-ABS material (43.7 MPa at peak). For the specimens made of 50PC-50ABS filament, the maximum load increased by up to 15.7% (R5 group, printed with 0.1 mm layer thickness). The highest tensile strength was obtained by samples made of 75PC-25ABS, obtaining an improvement of 32.3% for R9 samples with a 0.1 mm layer height. Thus, it can be observed that besides PC-ABS ratios, the layer thickness plays a significant role load capacity undertaken of the samples.

Regarding the strain, compared to the PC–ABS blend benchmark (6.4% elongation at peak), the samples made of the 25PC-75PC showed a decrease in the maximum elongation before the peak. The best results were obtained by the R1 set of samples (printed with 0.1 mm layer thickness), having a decrease of 11.9%. On the other hand, the specimens made of 50PC-50ABS material displayed an increase in elongation at peak. The best result was obtained for the R6 group (with 0.15 mm layer height) with 16.0% improvement. The last group of samples, printed with the 75PC-25ABS material, showed an increase of 28.3% for the R7 set of specimens with a 0.15 mm layer thickness.

Overall, besides the comparison with the PC–ABS blend reference material, it can be observed that most of the results of the experimental run lay between the stress and strain values of the materials used for 3D printing the core-shell filaments (Figure 7).

### 3.3. Variables Effects

The responses obtained from the experiments were analysed using a graphical representation of the main effects and an analysis of the variance of average tensile properties. Interaction effects between controlled variables were ignored as they were minimal. The response analysis helped identify the variables that had the most significant influence over the tensile strength of the specimens.

**Figure 8:**
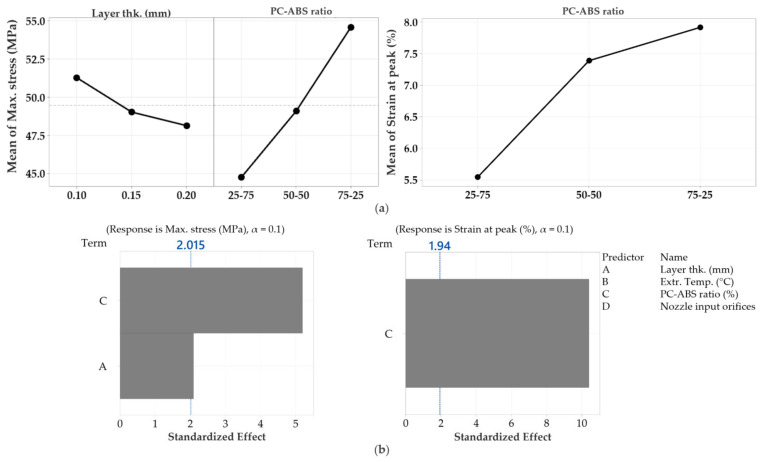
(**a**) Main effects plots of the variables. (**b**) Pareto chart of the standardised effects.

Overall, for both responses, stress and strain, the main effect plots showed that the PC–ABS ratio had the most significant influence over the tensile properties of the specimens (Figure 8).

The regression analysis was performed with a confidence level of 90% for both responses with the forward selection method to exclude the insignificant factors. For maximum stress, the regression equation is expressed as a function of materials ratios and layer thickness (Equation (1)) and strain as a function of materials ratios (Equation (2)),

Stress (MPa) = 54.17 − 31.3 × Lt − 4.711 × (25–75) − 0.388 × (50–50) + 5.099 × (75–25)(1)

Strain (%) = 6.9491 − 1.404 × (25–75) + 0.438 × (50–50) + 0.966 × (75–25)(2)

where Lt is the Layer thickness in mm, and (25–75), (50–50), and (75–25) are the PC–ABS ratios.

### 3.4. Model Validation

In order to validate the regression models, three supplementary trials were conducted, which included all materials ratios, printed at a 0.2 mm layer thickness at 260 °C, and with a regular nozzle. All resulting values fit the confidence and prediction intervals with a confidence level of 95%. The prediction data and results are available in Table 3.

## 4. Discussion

For the experimental setup, it was considered that the nozzles’ geometry and extrusion temperature could significantly influence the material flow behaviour and homogeneity. As a result, regardless of the PC–ABS ratios, nozzle input geometry, extrusion temperature, or layer height, the fracture zones showed the same pattern, characterised by a PC core and an ABS envelope similar to the initial core-shell structure of the 3D printed filaments (Figure 4, Figure 5 and Figure 6). These facts, correlated with the microscope analysis, showed that the filaments’ core-shell profile is maintained during the 3D printing process.

Except for R2 and R3 printing setups, which registered a lower tensile strength, the average strength and strain fit into the limits of materials used for printing the core-shell filament (Figure 7). Therefore, we assumed extrusion inconsistency or improper layer fusing affected R2 and R3 samples’ strength.

Peng et al. (2019) studied the impact and tensile properties of core-shell filaments of a PC–ABS core and an LDPE and HDPE shell produced through a normal extrusion process [15]. The results showed that the samples’ behaviour transitioned from brittle in the PC-ABS blend to ductile with the core-shell filament. Furthermore, similar properties were obtained for the 25PC-75ABS and 50PC-50ABS samples.

Ai et al. (2021) continued the previous work [12] by studying the dimensional accuracy of core-shell filaments of PC, cPC, or PC-ABS core and HDPE shell produced via conventional extrusion. The study highlights that the extrusion temperature does not significantly influence the tensile properties of the core-shell filaments [17]. This statement confirms the result of the regression equations (Equations (1) and (2)).

Producing filaments via 3D printing could be a potential solution in designing and testing new material blends at affordable prices but is not free of limitations and risks.

The first limitation is related to the maximum quantity of filament that can be produced in a printing process. It is dependent on printer build area (i.e., for Ultimaker 3, the maximum printable filament length was ≈8 m), and depending on the material ratios and feature size (e.g., shell thickness), smaller nozzles can be necessary (i.e., 75PC-25ABS was printed with a 0.25 mm nozzle), resulting in a higher printing time (i.e., ≈15 h).

**Figure 9:**
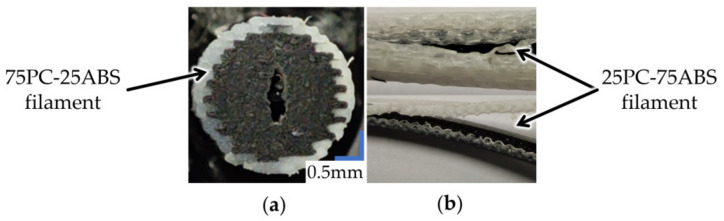
Possible defects of 3D printed filaments. (**a**) Gaps inside the 3D printed filament. (**b**) Shell detach during the printing.

Although the resulting average diameter was satisfactory (2.85 ± 0.1 mm), filament diameter could be affected during the brim removal. Another possible issue is print gaps that could appear in the filament structure (Figure 9a). As a precaution, an extrusion flow of ≈105% should be adopted to avoid the under-extrusion during printing.

During the printing process, shell detaching from the core (Figure 9b) can lead to filaments sticking in the printer Bowden tube. This defect may occur if the feeding mechanism presses the filament too hard.

Further research must be undertaken to characterise the performance of these materials for impact and bending strength, along with increasing the extrusion temperature to the upper limit of the used PC material (i.e., 270 °C).

## 5. Conclusions

This work studied a different approach to producing core-shell filaments for research purposes. Printable core-shell filaments with varying ratios of PC and ABS were made using a multi-material 3D printer. Specimens for tensile strength tests were produced with the resulting filaments. Other parameters such as nozzle input geometry, extrusion temperature, and layer thickness were considered to study the mixing degree of the core-shell filaments’ materials. Interestingly, regardless of these, the aspect of the core-shell filament was maintained throughout the entire printing process. Pure ABS samples were characterised by high ductility and low tensile stress, and PC specimens by brittleness and high load capacity. Thus, by combining them in the shape of a core-shell filament, it was possible to print samples with results between the limits of the materials. Higher content of ABS increases the specimens’ ductility but lowers the strength, and a higher content of PC makes the samples more brittle but increases the load resistance. Therefore, the FFF 3D printing process could be a convenient solution to explore and design new materials with a specific structure made of similar or dissimilar materials for research purposes.

## Figures and Tables

**Table 1:** Process variable of the Taguchi L9 matrix.

Variable/Level	Level 1	Level 2	Level 3
(1) PC–ABS ratios (%)	25–75	50–50	75–25
(2) Extrusion temperature (°C)	250	255	260
(3) Nozzle input orifices	Simple	Double	Triple
(4) Layer thickness (mm)	0.1	0.15	0.2

**Table 2:** Constant process parameters and their levels.

Parameter	Value	Parameter	Value
(1) Nozzle diameter (mm)	0.4	(10) Retraction distance (mm)	8
(2) Bed temperature (°C)	100	(11) Retraction speed (mm/s)	30
(3) Deposition speed (mm/s)	30	(12) ^2^ No. top layers	5–7–10
(4) First layer speed (mm/s)	15	(13) ^2^ No. bottom layers	5–6–10
(5) Line width (mm)	0.43	(14) Infill (%)	0
(6) Raster width (mm)	0.4	(15) Brim	Yes
(7) No. of walls	6	(16) Brim width (mm)	3
(8) ^1^ Material flow (%)	105	(17) Closed environment	Yes
(9) Fan speed (%)	0	(18) Bed temperature for print removal (°C)	30

^1^ (8) The material flow was changed to compensate for the 3D printed core-shell filament diameter inconsistency, ^2^ (12) No. of top and (13) No. bottom layers are influenced by the layer thickness presented in Table 1.

**Table 3:** Prediction and results for stress and strain.

**Setting**		**Prediction**			
**Variable**	**Value**	**Fit (MPa)**	**SE ^1^ Fit**	**95% CI ^1^ (MPa)**	**95% PI ^1^ (MPa)**
PC–ABS ratio (%)	25–75	43.2	1.29	(37.4, 46.5)	(37.4, 49.0)
	50–50	47.5		(41.7, 53.3)	(41.7, 53,3)
	75–25	53.0		(49.7, 58.8)	(47.2, 58.8)
Result	25–75	Average (MPa)	41.7	Standard deviation	1.6
	50–50		50.0		1.0
	75–25		54.2		1.5
**Setting**		**Prediction**			
**Variable**	**Value**	**Fit (%)**	**SE ^1^ Fit**	**95% CI ^1^ (%)**	**95% PI ^1^ (%)**
PC-ABS ratio (%)	25–75	5.5	1.38	(5.2, 5,9)	(4.9, 6.2)
	50–50	7.4		(7.0, 7,7)	(6.7, 8,1)
	75–25	7.9		(7.6, 8.6)	(7.2, 8.6)
Result	25–75	Average (%)	5.4	Standard deviation	0.1
	50–50		7.5		0.1
	75–25		7.9		0.2

^1^ with SE—Standard Error; CI—Confidence Interval; PI—Prediction Interval.

## Data Availability

The data presented in this study are available on request from the corresponding author.
